# Functional crosstalk between mTORC1/p70S6K pathway and heterochromatin organization in stress-induced senescence of MSCs

**DOI:** 10.1186/s13287-020-01798-1

**Published:** 2020-07-13

**Authors:** Hailong Liu, Biao Huang, Shaolong Xue, Kin Pong U, Lai Ling Tsang, Xiaohu Zhang, Gang Li, Xiaohua Jiang

**Affiliations:** 1 Key Laboratory for Regenerative Medicine of the Ministry of Education of China, School of Biomedical Sciences, Faculty of Medicine, The Chinese University of Hong Kong, Hong Kong, SAR People’s Republic of China; 2 Shenzhen Research Institute, The Chinese University of Hong Kong, Shenzhen, People’s Republic of China; 3 Department of Orthopaedics & Traumatology, Faculty of Medicine, The Chinese University of Hong Kong, Hong Kong, SAR People’s Republic of China; 4 Sichuan University-The Chinese University of Hong Kong Joint Laboratory for Reproductive Medicine, West China Second University Hospital, Sichuan University, Chengdu, 610041 Sichuan People’s Republic of China

**Keywords:** MSC senescence, mTORC1/p70S6K, Heterochromatin, Aging

## Abstract

**Background:**

Stem cell senescence has been proposed as one of the major drivers of aging, and MSC senescence contributes to aging-related diseases. Activation of mTORC1 pathway and heterochromatin organization have been characterized as two characteristics of senescent cells; however, whether mTORC1 pathway interacts with heterochromatin organization and contributes to MSC senescence remains unknown. In this study, we investigated the interaction between heterochromatin organization and mTORC1/p70S6K pathway in stress-induced MSC senescence.

**Methods:**

The stress-induced senescence models were established in human umbilical cord-derived MSCs by doxorubicin (Dox) or H_2_O_2_. Cellular senescence was evaluated by β-Gal activity, upregulation of cell cycle suppressor genes, and expression of SASP. Activation of heterochromatin organization and mTORC1 pathway was determined by Western blot and immunofluorescent staining. A D-galactose (D-Gal)-induced aging model was established in rats to evaluate the crosstalk between heterochromatin and mTORC1 pathway in vivo.

**Results:**

We found that heterochromatin organization was provoked at the early stage of Dox- or H_2_O_2_-induced senescence. Disruption of heterochromatin organization led to robust DNA damage response and exacerbated cellular senescence. Suppression of mTORC1/p70S6K pathway by either rapamycin or p70S6K knockdown promoted heterochromatin organization and ameliorated Dox- or H_2_O_2_-induced DNA damage and senescence. In contrast, direct activation of mTORC1 by MHY1485 impaired heterochromatin organization and aggravated stress-induced senescence. Moreover, concomitant activation of mTORC1 pathway and heterochromatin organization was found in D-galactose-induced osteoporosis model in rats. Rapamycin alleviated cellular senescence and promoted heterochromatin organization in BMSCs derived from D-galactose-treated rats.

**Conclusions:**

Altogether, our study indicates the existence of a complex interplay between the mTORC1/p70S6K pathway and the heterochromatin organization during stress-induced MSC senescence, with important implications for the understanding of aging as well as for its prevention and treatment.

## Background

Cellular senescence is a highly dynamic, multi-step process, during which the properties of senescent cells continuously evolve and diversify [1]. Senescent cells show marked changes in morphology including enlarged size, irregular cell shape, multiple nuclei, and prominent stress fibers that are accompanied by metabolic shift, accumulation of DNA damage, and a failure of autophagy [2]. Irreversible cell cycle arrest is an indicator of senescence, as senescent cells gradually exit from the cell cycle while preserving cell viability. In addition, senescent cells secrete pro-inflammatory and pro-oxidant signals recognized as senescence-associated secretory phenotype (SASP), which are involved in various biological processes, such as inflammation and tumorigenesis [3]. As a result of these mechanisms, senescent cells steadily accumulate with age and contribute to aging-related diseases, whereas clearance of senescent cells improves aging-related disorders and prolongs life span [4].

In recent years, accumulating evidence shows that persistent activation of the growth-promoting mammalian target of rapamycin complex 1 (mTORC1) pathway plays a critical role in cellular senescence and organismal aging [5, 6]. The activation of mTORC1 phosphorylates two major downstream targets, ribosomal protein S6 kinase (p70S6K), and eukaryotic initiation factor 4E-binding protein 1 (4E-BP1). Upon its phosphorylation, p70S6K regulates cell growth and proliferation, as well as protein synthesis [7]. The association between mTORC1 and organismal aging is demonstrated by various studies showing that inhibition of mTORC1 with rapamycin significantly extends lifespan in all studied model organisms including mammals [8–12]. The first indication that mTORC1 is a regulator of lifespan originated from studies with the nematode *Caenorhabditis elegans* [10] and the fruit fly *Drosophila melanogaster* [12]. The notion was further strengthened by the finding that rapamycin prolonged the lifespan of genetically heterogeneous mice by about 10–18% depending on sex [13]. Similarly, deficiency of mTORC1 substrate S6K1 increased lifespan in female but not in male mice [11]. Altogether, these findings suggest an evolutionary conserved role of mTORC1 in regulating aging and longevity. While the exact mechanism by which mTORC1 inhibition protects from cellular senescence and organismal aging remains unclear, the anti-aging effects have been associated with reduced accumulation of reactive oxygen species [14] and DNA damage [15], decreased secretion of SASP [16], switch of cellular energy metabolism [6], and reduced expression of tumor suppressors such as p16^INK4A^ [17].

Another well-recognized feature of senescent cells is the extensive spatial rearrangement of heterochromatin, forming nuclear structures known as senescence-associated heterochromatic foci (SAHF) [18]. SAHF consists of high-mobility group A and dense, repressive chromatin foci which are enriched by H3K9me3, heterochromatin protein 1, and macro H_2_A. While the formation of SAHF was most prominent in oncogene-induced senescence and thought to be essential for the maintenance of senescent state [19], SAHF formation and senescence are not always coupled [20]. For instance, cellular models of organismal aging such as cells from Hutchinson-Gilford progeria syndrome (HGPS) and Werner syndrome patients show a decrease or loss in heterochromatin marks and are devoid of SAHF structure [19, 21, 22]. Indeed, a recent study clearly showed that heterochromatin disorganization led to premature senescence in *WRN*-deficient mesenchymal stromal cells (MSCs) [23]. In addition, human MSCs derived from older individuals display reduced heterochromatin marks, which support the crucial role of heterochromatin stability in human aging [20, 23]. Altogether, these findings suggest that the unique and organized change of heterochromatin landscape may function as a protective mechanism against cellular senescence. However, the molecular mechanisms underlying the heterochromatin organization process in cellular senescence remain unclear.

With high capacity of self-renewal and differentiation, MSCs play a key role in maintaining tissue homoeostasis and regeneration ability. Deterioration of MSC function has been recognized as an important hallmark of organismal aging [24–27]. On the other hand, the possibility of retarding senescence and extending stemness properties of in vitro expanded MSCs is particularly relevant to regenerative medicine, as replicative senescence limits the number of cells required for clinical application [28]. Thus, it is imperative for us to understand the mechanisms underlying MSC senescence. Given the critical role of mTORC1-mediated signaling and heterochromatin organization in senescence regulation, we undertook the present study to determine whether mTORC1 pathway cross talks with heterochromatin organization in stress-induced MSC senescence.

## Methods

### Isolation and characterization of MSCs

The use of the human umbilical cord for MSC isolation was approved by Joint CUHK-NTEC Clinical Research Ethics Committee (ethical approval code: CRE-2011.383 and CRE-2015.018). Reagents for cell culture were purchased from Gibco, Life Technologies (Carlsbad, CA). The isolation was conducted, and cells were cultured in knockout DMEM (KO-DMEM, Cell Treatment Therapy, CTS, grade) supplemented with 10% FBS (fetal bovine serum, Gibco, Life Technologies), 1% P/S (CTS), and 1% glutamax (CTS) as described in our previous study [20]. The MSCs were characterized according to ISCT (2006) minimal criteria. The cells were initially seeded at 100,000/cm^2^ and washed with PBS the day after. After cell confluence reached 90%, hUC-MSCs were passaged using 0.05% trypsin and re-plated at 10,000 cells/cm^2^ in T175 or T75 flasks (Corning). Three hUC-MSC lines (hUC009, hUC011, hUC013) were used in our study (p2-p9), and proliferation was assessed until passage 9. The population doubling time (PDT) was calculated using the following equation: time/log_2_(harvested cells/seeded cells). While the first passage PDT of hUC011 was significantly longer compared to the other two lines (hUC009 66 ± 3.2 h, hUC011 75 ± 7.8 h, hUC013 59 ± 11 h), the subsequent PDT between passage 5–9 was comparable among the three lines. Phenotypic characterization of the hUC-MSC lines was conducted at passage 3–4. We used the hUC-MSCs at passage 5–9 for the functional studies.

For primary rat BMSCs isolation, BM was harvested and pooled by flushing the femurs and the tibia of rats with α-MEM supplemented with 10% FBS and 1% penicillin-streptomycin [20]. The cells were initially seeded at 100,000/cm^2^ and washed with PBS the day after. Adherent cells were expanded when cells reached 80–90% confluence and re-plated at 5000 cells/cm^2^ in T175 or T75 flasks. In total, four BMSC lines were established from control rats and four BMSC lines were established from D-galactose-treated rats. Phenotype analysis of surface markers was done at passage 2–3 based on two positive markers CD90 and CD54 and two negative markers CD45 and CD34 by FACS. We used the rBMSCs at passage 4–5 for the following experiments. The PDT or rBMSCs from control and D-galactose-treated rats were evaluated at passage 3–5. On average, the PDT of rBMSCs at passage 3 is 89 ± 6.7 h.

### Cell treatment in vitro

For doxorubicin treatment, early passage hUC-MSCs (p5-p7) were treated with doxorubicin (Selleckchem, Houston, TX, USA) at 1 × 10^−8^ M for 24 h. After 24 h treatment, the cells were washed with PBS and continued to culture for 0 h, 3 h, 9 h, 12 h, 24 h, 48 h, and 96 h. At the end of the experiments, RNA samples and protein samples were collected for real-time PCR, Western blot, and immunofluorescent staining. For H_2_O_2_ treatment, hUC-MSCs (p5-p7) were treated with H_2_O_2_ (300 nM) for 3 h, washed with PBS, and continued to grow for another 0 h, 3 h, 9 h, 12 h, 24 h, 48 h, and 96 h. At the end of the experiments, RNA samples and protein samples were collected for real-time PCR, Western blot, and immunofluorescent staining. Alternatively, cells were fixed with formaldehyde for β-Gal staining. For rapamycin or MHY1485 treatment, hUC-MSCs of passage 8–9 were treated with rapamycin (5 nM) or MHY1485 (2 nM) for 1 h and then subjected to doxorubicin (1 × 10^−8^ M) for 24 h or H_2_O_2_ (300 nM) for 3 h. hUC-MSCs of passage 8–9 were treated with Chaetocin (10 nM) for 48 h and then subjected to doxorubicin for 24 h.

### siRNA transfection

siRNA pools targeting *RPS6KB1* were purchased from GenePharma (Shanghai, China). hUC-MSCs were transfected with siRNAs using Lipofectamine® 3000 Transfection Reagent (Life Technologies). Forty-eight to 72 h after transfection, the knockdown efficiency was determined by quantitative real-time PCR or Western blot. siRNA sequences used in this study are *RPS6KB1* 5′GCAAAGAUCUCAUGGGCUUTT, 5′AGCCCAUGAGAUCUUUGCTT, negative control (si-NC), 5′CGUGGGUGGAUGCAUGGAUTT.

### β-Gal staining

We conducted the β-Gal staining using a β-Gal Staining assay kit (Cell Signaling Technology, Danvers, MA, USA) following the manufacturer’s protocol. Briefly, cells were washed with PBS and fixed with 1× Fixative Solution for 10 min at room temperature. After washing, the cells were incubated at 37 °C with fresh β-Galactosidase Staining Solution [pH 6.0] for 24 h. The number of SA-*β*-gal-positive cells was determined in 10 randomly chosen fields, and a total of at least 200 cells from each sample were counted. The experiment was repeated at least three times.

### Clonogenic assay

Two thousand hUC-MSC cells were seeded in six-well cell culture plates and cultured for 7 days. The cells were fixed with 100% methanol for 15 min and stained with 1% crystal violet aqueous solution for 5 min.

### RNA extraction and real-time PCR

Total RNA was extracted using TRIzol reagent (Life Technologies). One microgram of RNA was used for reverse transcription using the high capacity reverse transcription kit (Applied Biosystems, Cat.No.00599192) following the manufacturer’s protocol. Real-time RT-PCR reactions were performed using the SYBR Green PCR kit (Takara, Kusatsu, Shiga, Japan) and a 7500 Fast Real-Time PCR System (Applied Biosystems, CA, USA). The primers used for specific genes are presented in Supplementary Table 1. The expression of the target genes was normalized to that of *GAPDH*. Relative gene expression was calculated using the 2^−ΔCT^ formula.

### Western blot

Cells were lysed and protein was extracted using RIPA (Pierce, Rockford, IL, USA) plus protease inhibitor cocktail (Thermo Fisher, Waltham, MA, USA), and protein concentrations were determined using the BCA assay (Bio-Rad, Richmond, CA, USA). Aliquots of protein lysates were separated on SDS-6, 8, 10, 12% polyacrylamide gels and transferred onto polyvinylidene difluoride (PVDF) membrane, which was blocked with 4% blotting-grade milk in TBST (20 mM Tris–HCl [pH 7.6], 137 mM NaCl, and 1% Tween 20). The membrane was then hybridized with the indicated primary antibodies followed by the corresponding secondary antibodies (1:5000) for 90 min at room temperature and then detected using ECL (GE). Finally, enhanced chemiluminescence (ECL) or ECL plus or ECL prime systems were used to detect the target bands. The antibodies used in this study are listed in Supplementary Table 2.

### Immunofluorescent staining

Cells seeded on 10 mm coverslips were fixed with 4% paraformaldehyde for 10 min and washed with PBS for three times. The slips were permeabilized with 0.1% Triton X-100 in PBS for 20 min and blocked with 3% BSA for 30 min. After that, cells were incubated with primary antibody at 4 °C overnight, washed, and incubated with secondary antibody at room temperature for 90 min. The Alexa Fluor Series from Invitrogen were used as secondary antibodies. Images were taken using a confocal system with inverted microscope (Olympus FV1000) and analyzed with FluoView 4.2a. For all quantitative analyses, images were collected using the same acquisition parameters to facilitate fluorescence intensity comparisons between groups. Cell fluorescence intensity (CTCF) was defined as area × (mean intensity − background intensity). For DNA damage foci analysis, all experiments were repeated three times, each experiment was set up for triplicates. Five fields per slide were analyzed under the microscope, and no less than 100 cells were analyzed for quantification data.

### D-galactose-induced rat aging model

Sixteen 4 month-old Sprague-Dawley male rats were used. They were maintained in an air-conditioned room with a controlled temperature of 24 ± 2 °C and humidity of 55 ± 15%, in a 12-h light/darkness cycle regulation, and were fed laboratory chow and water ad libitum. All animal experiments were conducted in accordance with the University Laboratory Animals Service Center’s guidelines with approval from the Animal Ethics Committee of the University. After an initial acclimatization of 1 week, SD male rats were randomly divided into two groups. The D-gal-treated group was injected with 125 mg/kg D-galactose dissolved in sterile 0.9% saline intra-peritoneally daily. The control group was injected with the same volume of 0.9% saline. After consecutive injection for 4 months, the animals were sacrificed and the tibia and femur were collected for further study.

### Micro-computed tomography (micro-CT) scanning

The rat femurs were scanned using a desktop preclinical specimen micro-computed tomography (uCT-35, Scanco Medical, Bassersdorf, Switzerland). In brief, the femurs were fixed with 4% paraformaldehyde for 24 h and scanned using microCT35 with a resolution of 8 mm isometric voxel at 70 kV and 114 mA. Three-dimensional (3D) reconstructions of mineralized tissues were performed by an application of a global threshold (211 mg hydroxyapatite/cm3), and a Gaussian filter (sigma = 0.8, support = 2) was used to suppress noise. The three-dimensional reconstructed images were used directly to quantify microarchitecture, and the morphometric parameters including bone volume fraction (BV/TV), trabecular number (Tb.N, 1/mm), trabecular separation (Tb.Sp, mm), and trabecular thickness (Tb. Th, mm) were calculated with the image analysis program of the micro-CT workstation (Image Processing Language v4.29d, Scanco Medical, Switzerland).

### Statistical analysis

Data were analyzed by GraphPad Prism 6. Data were expressed as the mean ± SEM. Differences in measured variables between two groups were assessed by using Student’s *t* tests. One-way or two-way ANOVA with relevant post hoc tests was used for multiple-group comparisons. Results were considered statistically significant at *p* < 0.05.

## Results

### Doxorubicin or H_2_O_2_ induces stress-induced premature senescence (SIPS) in hUC-MSCs

**Fig. 1:**
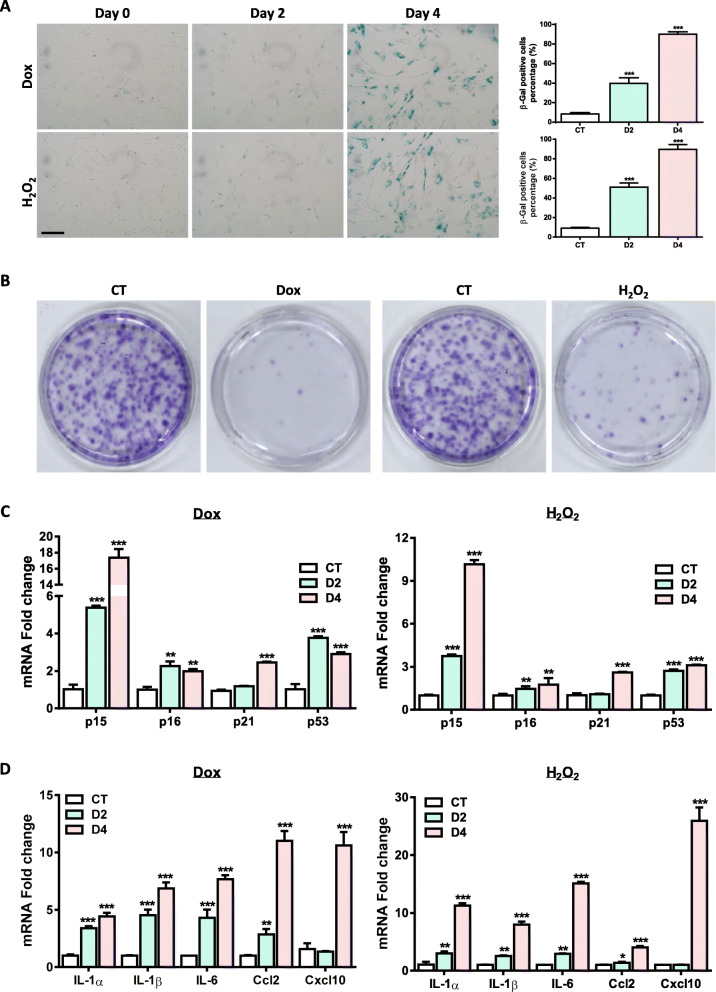
Doxorubicin or H_2_O_2_ induces hUC-MSC senescence. 2 × 10^5^ hUC-MSCs (p5-p7) were seeded in 6-well plates and treated with Dox (10^−8^ M) for 24 h or H_2_O_2_(300 nM) for 3 h, then washed with PBS and grown for another 2–4 days. After that, the cells were subjected to various analyses. **a** β-Gal staining was conducted at day 2 or day 4 in Dox- or H_2_O_2_-induced hUC-MSCs (scale bar = 100 μm). The quantification of β-Gal staining is shown on the right. **b** Two thousand hUC-MSCs were seeded in 6-well plates and treated with Dox (10^−8^ M) for 24 h or H_2_O_2_(300 nM) for 3 h, then washed with PBS and grown for another 7 days. **c** qRT-RCR analysis of cell cycle-related genes in Dox- or H_2_O_2_-induced hUC-MSCs; cells were collected at day 2 or day 4. **d** qRT-PCR analysis of senescence-associated secretory phenotype (SASP) genes in Dox- or H_2_O_2_-induced hUC-MSCs; cells were collected at day 2 or day 4. Data are presented as the mean ± SEM. **p < 0.01;***p < 0.001 by Student *t* test

We used doxorubicin (Dox) or H_2_O_2_ to induce senescence in this study, which represents genotoxic stress- or oxidative stress-induced cellular senescence, respectively. In accordance with previous reports, short period and low concentration of Dox or H_2_O_2_ treatment lead to MSC senescence (Fig. 1a–d). hUC-MSCs gradually developed major phenotypes of cellular senescence, including increased number of senescence-associated–β-galactosidase (SA-β-gal)–positive cells and loss of proliferative potential 48 to 96 h after the end of Dox or H_2_O_2_ treatment (Fig. 1a, b). In addition, the expression levels of cell cycle inhibitors including *p15*, *p16*, *p53*, and *p21* were consistently increased in Dox- or H_2_O_2_-treated hUC-MSCs (Fig. 1c). SASP represents another characteristic shared by senescent cells. Our real-time PCR results showed that the expression levels of the major members of SASP were markedly increased along with the cellular senescence process induced by Dox or H_2_O_2_ (Fig. 1d). Consistently, the expression levels of SASP members reached their peak at day 4 after Dox or H_2_O_2_ withdrawal. This data is in line with the β-gal staining and colony formation data that Dox or H_2_O_2_ induces a robust cellular senescence phenotype in hUC-MSCs predominantly at day 4 from the end of Dox or H_2_O_2_ treatment, at which more than 80% of the hUC-MSCs are positive for β-gal and cell proliferation is drastically compromised.

### Rapamycin alleviates SIPS-associated changes induced by Dox or H_2_O_2_

**Fig. 2:**
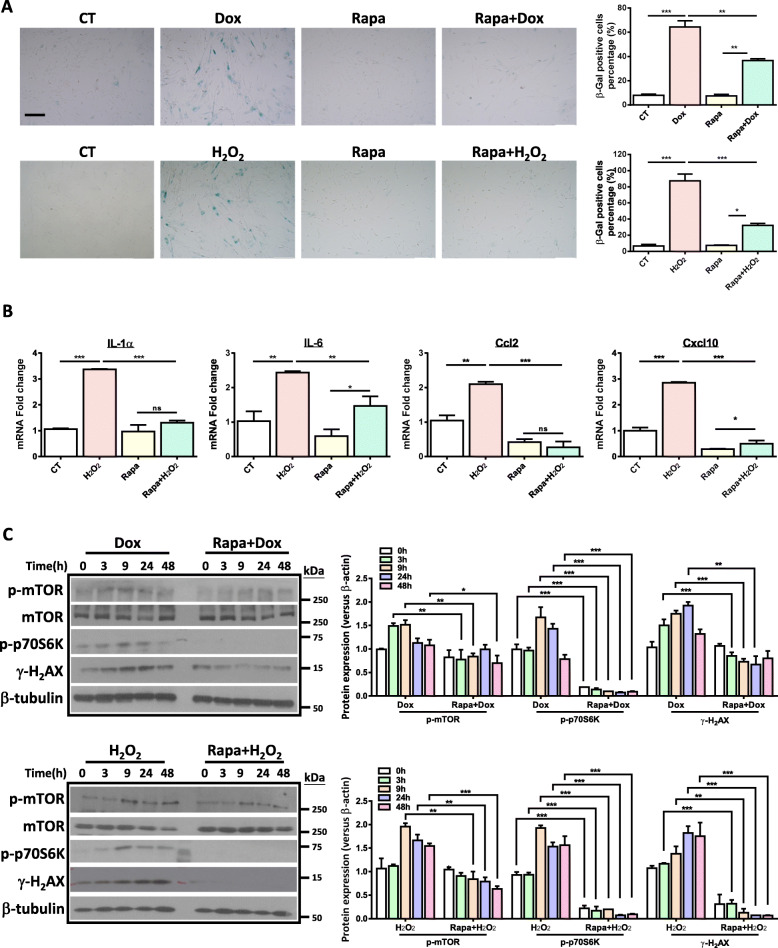
Rapamycin alleviates stress-induced senescence of hUC-MSCs. 2 × 10^5^ hUC-MSCs (p8-p9) were seeded in 6-well plates and treated with Dox or H_2_O_2_ with or without rapamycin. **a** Cells were collected 4 days after Dox or H_2_O_2_ treatment. β-Gal staining showing that rapamycin (5 μM) alleviates Dox- or H_2_O_2_-induced hUC-MSC senescence (scale bar = 100 μm). The quantification data is shown on the right. Data are presented as the mean ± SEM. **p < 0.01;***p < 0.001 by one-way ANOVA with Tukey’s post hoc test. **b** Cells were collected 2 days after Dox or H_2_O_2_ treatment. qRT-PCR analysis shows rapamycin reduces H_2_O_2_-induced SASP. Data are presented as the mean ± SEM. *p < 0.05, **p < 0.01;***p < 0.001 by one-way ANOVA with Tukey’s post hoc test. **c** Representative Western blot shows that rapamycin reduces the activation of p70S6K and alleviates stress-induced DNA damage response. Quantification data is shown on the right. Data are achieved from three independent experiments. *p < 0.05; **p < 0.01;***p < 0.001 by two-way ANOVA with Bonferroni post hoc test first, followed by paired Student *t* test

To explore the possible influence of mTORC1 pathway in hUC-MSC senescence, we pretreated hUC-MSCs with rapamycin before the induction of Dox or H_2_O_2_, and then analyzed changes in senescence phenotypes. Our results showed that rapamycin significantly decreased the percentage of β-Gal-positive cells in the two senescence models (Fig. 2a). Moreover, rapamycin prevented H_2_O_2_-induced expression of SASP (Fig. 2b). To further study the molecular mechanism underlying the effect of rapamycin on cellular senescence, we determined the expression of p-mTOR and its downstream target p70S6K in the two senescence models pretreated with rapamycin. We found that the rapid activation of mTORC1 and p70S6K by either Dox or H_2_O_2_ was dramatically suppressed by rapamycin (Fig. 2c). Since both Dox and H_2_O_2_ can induce DNA damage_**,**_ we also determined whether rapamycin assuaged cellular senescence via relieving DNA damage accumulation. Interestingly, while DNA damage accumulated with the progression of senescence in Dox- or H_2_O_2_-treated MSCs, rapamycin dramatically reduced DNA damage accumulation starting from 3 h after the treatment of Dox or H_2_O_2_ as indicated by the decreased expression level of γ-H_2_AX, which is a well-established marker for double-strand DNA breaks (Fig. 2c). Altogether, these results suggest that rapamycin mitigates Dox- or H_2_O_2_-induced cellular senescence of hUC-MSCs, possibly via alleviating the DNA damage accumulation.

### mTORC1/p70S6K pathway regulates SIPS in hUC-MSCs

**Fig. 3:**
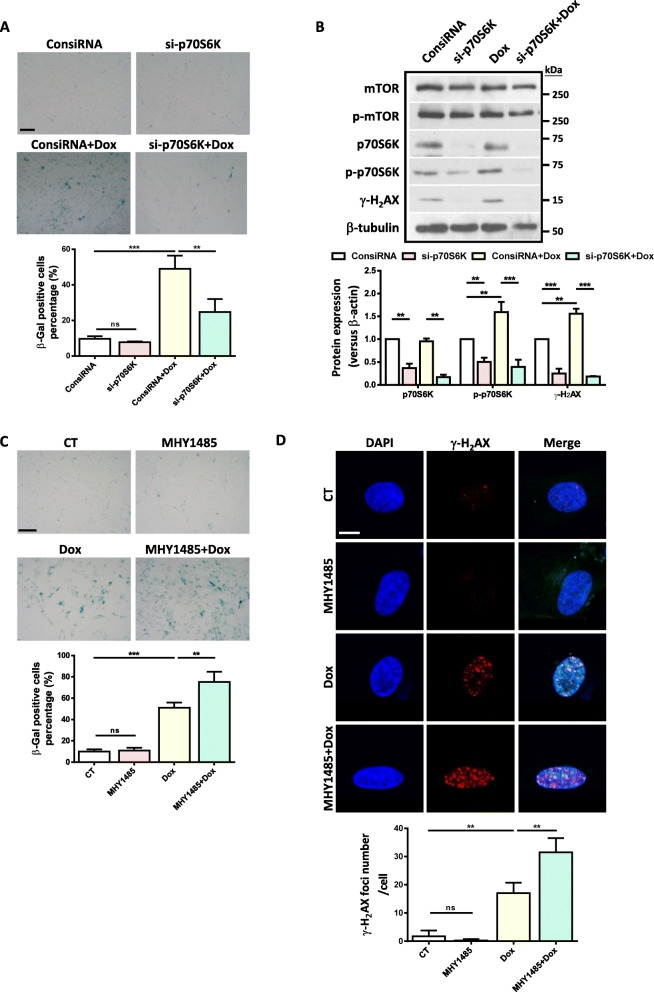
mTORC1 regulates stress-induced senescence via p70S6K. **a** β-gal staining of control siRNA- or p70S6K-siRNA-treated hUC-MSCs in the presence or absence of Dox. 1 × 10^5^ hUC-MSCs (p5-p7) were seeded in 6-well plates and transfected with siRNAs. Twenty-four hours later, the cells were treated with Dox and collected 2 days afterward. Quantification data is shown below. Data are presented as the mean ± SEM. **p < 0.01;***p < 0.001 by one-way ANOVA with Tukey’s post hoc test. **b** Representative Western blot showing that knockdown of p70S6K alleviates Dox-induced DNA damage response. Cells were treated with Dox and collected 24 h later. Data are presented as the mean ± SEM. **p < 0.01;***p < 0.001 by one-way ANOVA with Tukey’s post hoc test. **c** The β-Gal staining shows that MHY1485 aggravates Dox-induced cellular senescence (scale bar = 100 μm). 2 × 10^5^ hUC-MSCs (p8-p9) were seeded in 6-well plates and treated with Dox with or without MHY1485. The cells were collected 2 days afterward. The quantification of β-Gal staining is shown below. Data are presented as the mean ± SEM. **p < 0.01;***p < 0.001 by one-way ANOVA with Tukey’s post hoc test. **d** 1 × 10^4^ hUC-MSCs (p8-p9) were seeded on coverslip and treated with Dox with or without MHY1485. The cells were collected 24 h afterward. Representative images and quantification of immunofluorescence staining of γ-H_2_AX in control or MHY1485-treated hUC-MSCs in the presence or absence of Dox (scale bar = 10 μm). Quantification is shown below, mean ± SEM of values from three independent experiments with triplicate wells analyzed on 6–8 cells/field from five different fields. **p < 0.01 one-way ANOVA with Tukey’s post hoc test

Given that rapamycin protects hUC-MSCs from stress-induced cellular senescence and rapamycin completely abolishes the activation of p70S6K in the presence of Dox or H_2_O_2_, it is plausible that p70S6K, the direct target of mTORC1, plays a crucial role in hUC-MSC senescence. Thus, we knocked down p70S6K using siRNAs to determine whether suppression of p70S6K exhibited the same effect as rapamycin did on hUC-MSC senescence. Our results showed that knockdown of p70S6K dramatically alleviated Dox- or H_2_O_2_-induced hUC-MSC senescence (Fig. 3a and Fig. S1). In addition, suppression of p70S6K reduced the basal and Dox-induced expression of γ-H_2_AX (Fig. 3b). To further demonstrate the causative role of the mTORC1 pathway on cellular senescence, we tried to evaluate the effect of direct activation of mTORC1 pathway on senescence. To do this, we pretreated hUC-MSCs with a direct activator of mTORC1, MHY1485, which has been shown to activate mTORC1 and downstream substrate [29]. Significantly, pretreatment with MHY1485 aggravated Dox-induced cellular senescence in hUC-MSCs (Fig. 3c). Next, we detected the degree of DNA damage using immunofluorescent staining. Our results showed that the number of γ-H_2_AX foci was significantly increased in the MHY1485 and Dox treatment group compared to the Dox-only group (Fig. 3d). Altogether, these results suggest that mTORC1/p70S6K pathway regulates stress-induced DNA damage and cellular senescence in hUC-MSCs.

### Heterochromatin organization protects hUC-MSC from Dox- or H_2_O_2_-induced DNA damage and cellular senescence

**Fig. 4:**
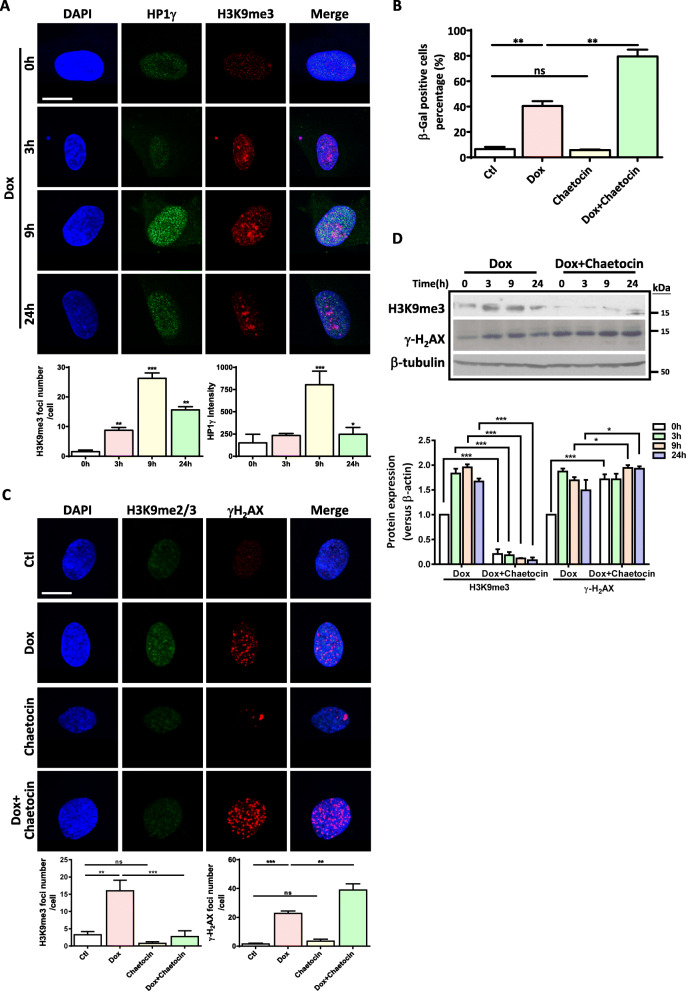
Heterochromatin organization protects hUC-MSCs from Dox- or H_2_O_2_-induced DNA damage and cellular senescence. **a** Representative photos and quantification of immunofluorescence staining of H3K9me3 and HP1γ in Dox-induced hUC-MSCs at different time points (scale bar = 10 μm). 1 × 10^4^ hUC-MSCs (p5-p7) were seeded on coverslip and treated with Dox. The cells were washed with PBS and incubated in the fresh media for 24 h. Quantification is shown below, mean ± SEM of values from three independent experiments with triplicate wells analyzed on 6–8 cells/field from five different fields. *p < 0.05; **p < 0.01;***p < 0.001 by Student’s *t* test. **b** β-gal staining indicates Chaetocin aggravates Dox-induced cellular senescence. 2 × 10^5^ hUC-MSCs (p5-p7) were seeded in 6-well plates and treated with Dox with or without Chaetocin. The cells were collected 2 days afterward. Data are presented as the mean ± SEM. *p < 0.05;**p < 0.01 by one-way ANOVA with Tukey’s post hoc test. **c** Representative images and quantification of immunofluorescence staining of H3K9me2/3 and γ-H_2_AX in control or Chaetocin-treated hUC-MSCs in response to Dox (scale bar = 10 μm). 1 × 10^4^ hUC-MSCs (p5-p7) were seeded on coverslip and treated with Dox with or without Chaetocin. The cells were collected 24 h afterward. Quantification is shown below, mean ± SEM of values from three independent experiments with triplicate wells analyzed on 6–8 cells/field from five different fields. **p < 0.01;***p < 0.001 by one-way ANOVA with Tukey’s post hoc test. **d** Representative Western blot image of H3K9me3 and γ-H_2_AX in Dox-induced hUC-MSCs senescence with or without Chaetocin. 2 × 10^5^ hUC-MSCs (p5-p7) were seeded in 6-well plates and treated with Dox with or without Chaetocin. The cells were collected at different time points. Quantification data is shown below. Data are achieved from three independent experiments. *p < 0.05; **p < 0.01;***p < 0.001 by two-way ANOVA with Bonferroni post hoc test first, followed by paired Student *t* test

Our previous study demonstrated that MSC replicative senescence was accompanied by a dynamic change of heterochromatin structure [20]. In consistence with the previous study, we found a dramatic loss of repressive heterochromatin marks at the late stage of SIPS when more than 80% of hUC-MSCs exhibited senescence phenotype (Fig. S2A and Fig. 1a). Noticeably, we also observed an induction of heterochromatin marks, such as H3K9me3 and HP1γ, at the early stage of SIPS starting from 3 h after Dox or H_2_O_2_ treatment at which only small amount of cells exhibited senescent features. Additionally, both the intensity of HP1γ and the number of H3K9me3 foci reached their peak at 9 h and decreased thereafter (Fig. 4a and Fig. S2B), indicating Dox or H_2_O_2_ induces a heterochromatin organization process at the early stage of SIPS. To further determine the role of heterochromatin organization in the early stage of SIPS, we asked the question of whether direct disruption of heterochromatin organization would affect the DNA damage accumulation and cellular senescence. We pretreated the hUC-MSCs with Chaetocin, a nonspecific inhibitor of H3K9 lysine methyltransferase, to block the stress-induced heterochromatin organization process and monitored the change of DNA damage and cellular senescence. Our results showed that Chaetocin treatment exacerbated Dox-induced hUC-MSC senescence (Fig. 4b and Fig. S2C). In addition, while Chaetocin alone did not have a significant effect on DNA damage, pretreatment with Chaetocin significantly increased the number of Dox-induced γ-H_2_AX foci (Fig. 4c). Consistently, our Western blot results demonstrated that Chaetocin pretreatment abolished the induction of heterochromatin marks, whereas augmented DNA damage response in hUC-MSCs treated with Dox (Fig. 4d). Collectively, these data demonstrate a protective role of heterochromatin organization, disruption of which leads to magnified DNA damage response followed by progressive senescence in response to stress.

### mTORC1/p70S6K pathway cross talks with heterochromatin organization in SIPS of hUC-MSCs

**Fig. 5:**
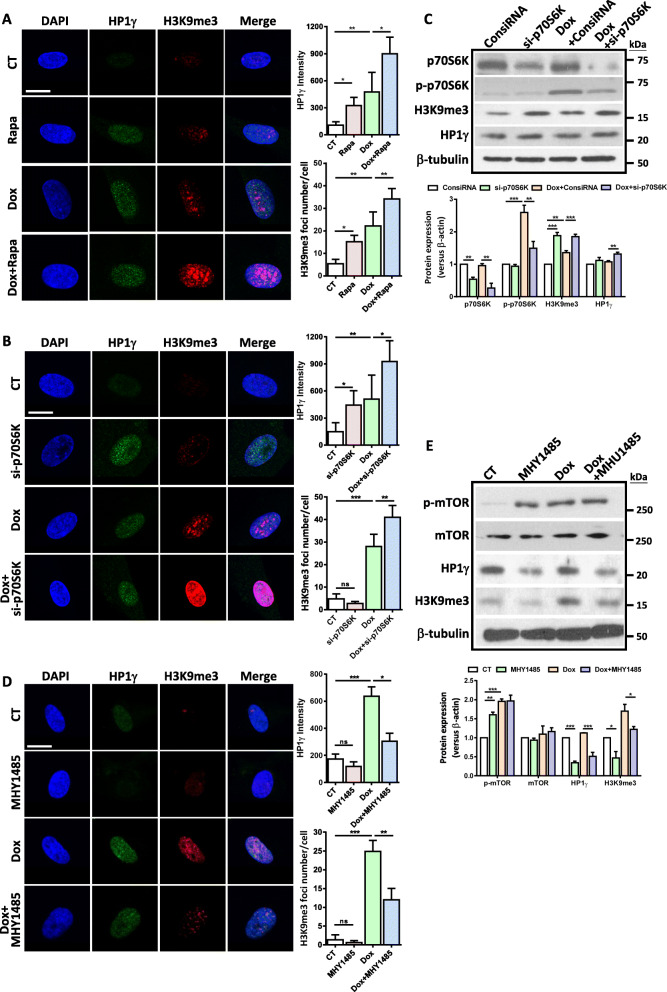
mTORC1 pathway cross talks with heterochromatin organization during stress-induced senescence. **a** 1 × 10^4^ hUC-MSCs (p8-p9) were seeded in coverslips and treated with Dox with or without rapamycin. Cells were collected after 24 h. Representative images and quantification of immunofluorescence staining of HP1γ and H3K9me3 in control or rapamycin-treated hUC-MSCs in response to Dox (scale bar = 10 μm). Quantification is shown on the right panel, mean ± SEM of values from three independent experiments with triplicate wells analyzed on 6–8 cells/field from five different fields.*p < 0.05; **p < 0.01 by one-way ANOVA with Tukey’s post hoc test. **b** 1 × 10^4^ hUC-MSCs (p5-p7) were seeded coverslips and transfected with siRNAs. Twenty-four hours later, the cells were treated with Dox and collected 1 day afterward. Immunofluorescent staining of heterochromatin marks H3K9me3 and HP1γ shows that knockdown of p70S6K increases the H3K9me3 foci and HP1γ intensity. Quantification is shown on the right panel, mean ± SEM of values from three independent experiments with triplicate wells analyzed on 6–8 cells/field from five different fields.*p < 0.05; **p < 0.01, ***p < 0.001 by one-way ANOVA with Tukey’s post hoc test. **c** Representative Western blot shows that knockdown of p70S6K increases the expression levels of H3K9me3 and HP1γ. Data are presented as the mean ± SEM. **p < 0.01; ***p < 0.001 by one-way ANOVA with Tukey’s post hoc test. **d** 1 × 10^4^ hUC-MSCs (p8-p9) were seeded in coverslips and treated with Dox with or without MHY1485. Cells were collected after 24 h. Representative images and quantification of immunofluorescence staining of HP1γ and H3K9me3 in control or MHY1485-treated hUC-MSCs in response to Dox (scale bar = 10 μm). Quantification is shown on the right panel, mean ± SEM of values from three independent experiments with triplicate wells analyzed on 6–8 cells/field from five different fields.*p < 0.05; **p < 0.01;***p < 0.001 by one-way ANOVA with Tukey’s post hoc test. **e** Representative Western blot shows that MHY1485 reduces the expression levels of H3K9me3 and HP1γ. Quantification data is shown below. Data are achieved from three independent experiments. *p < 0.05; **p < 0.01;***p < 0.001 by one-way ANOVA with Tukey’s post hoc test

We have shown that both mTORC1/p70S6K pathway and heterochromatin organization are involved in SIPS of hUC-MSCs. Our next question is whether these two divergent mechanisms cross talk with each other in the progression of SIPS. To do this, we manipulated the mTORC1/p70S6K pathway using various methods and determined the change of heterochromatin organization in hUC-MSCs. We first pretreated hUC-MSCs with rapamycin and monitored change of heterochromatin in response to Dox. Our results showed that rapamycin significantly increased the expression of heterochromatin marks at both basal level and upon Dox or H_2_O_2_ treatment (Fig. 5a and Fig. S3). In addition, knockdown of p70S6K promoted the induction of heterochromatin organization in response to Dox (Fig. 5b). In consistence with this result, our Western blot result showed that knockdown of p70S6K increased the protein expression levels of H3K9me3 and HP1γ (Fig. 5c). Next, we investigated whether direct activation of mTORC1 pathway could influence heterochromatin organization. Interestingly, we found that MHY1485 significantly suppressed the induction of HP1γ and H3K9 foci by Dox (Fig. 5d). Consistently, Western blot results showed that the expression levels of H3K9me3 and HP1γ were downregulated in the MHY1485-treated group with or without Dox (Fig. 5e). These results indicate that mTORC1/p70S6K pathway negatively regulates heterochromatin formation, which affects the progression of SIPS-induced DNA damage accumulation and senescence.

### Interplay between mTORC1 pathway and heterochromatin organization in D-Gal-induced bone aging

**Fig. 6:**
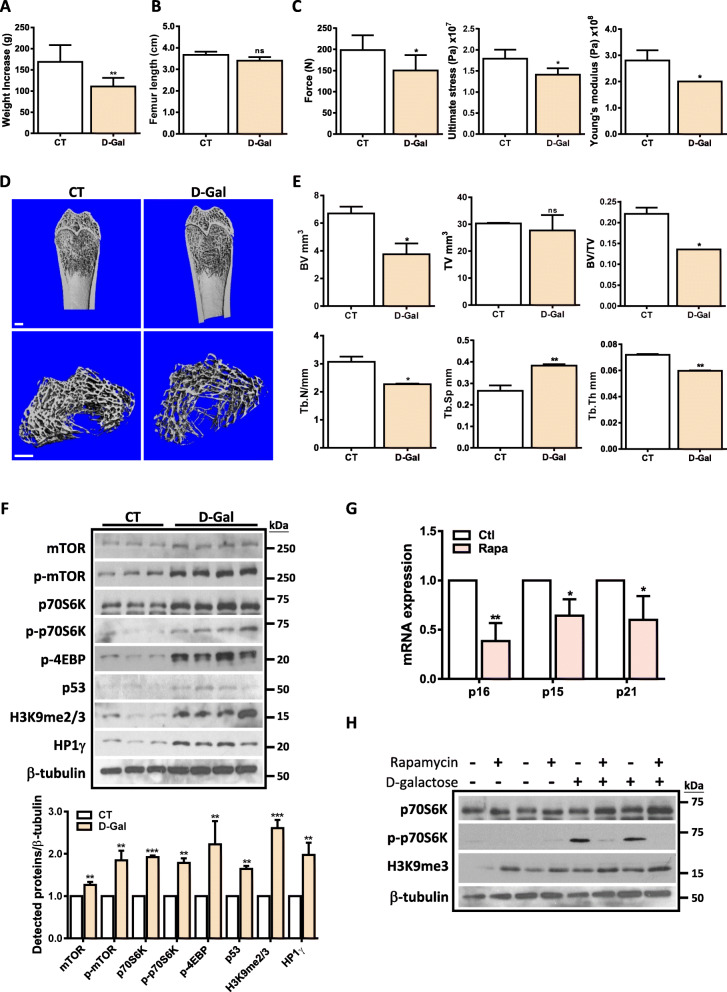
Activation of mTORC1 pathway and heterochromatin organization in D-Gal-induced rat aging. SD male rats were randomly divided into two groups. The D-gal-treated group was injected with 125 mg/kg D-galactose dissolved in sterile 0.9% saline intra-peritoneally daily. The control group was injected with the same volume of 0.9% saline. After consecutive injection for 4 months, the animals were sacrificed and the tibia and femur were collected for further study. **a** Weight increment of rats of each group (*n* = 8) at the end of experiments, **p < 0.01 by Student *t* test. **b** Femur length of rats of each group (*n* = 4). **c** Test of maximum load, ultimate stress, and Young’s modulus of femurs from each group (*n* = 8), *p < 0.05 by Student *t* test. **d** Representative 3D image of femurs (*n* = 4) and representative 3D image of femur regions of interest (the range between 300 and 450 slices below the growth plate). **e** Quantification of morphometric parameters including bone volume (BV), trabecular volume (TV), bone fraction (BV/TV), trabecular number (Tb.N, 1/mm), trabecular separation (Tb.Sp, mm), and trabecular thickness (Tb, Th, mm) are shown. *p < 0.05; **p < 0.01 by Student *t* test (*n* = 4). **f** Western blot analysis in BMSCs (p3-4) isolated from rats of each group (CT: *n* = 3, D-gal, *n* = 4). Quantification data is shown below, *p < 0.05; **p < 0.01;***p < 0.001 by Student *t* test. **g** 1 × 10^6^ primary BMSCs derived from control or D-Gal treated rats (p3-p5) were treated with rapamycin for 48 h. RT-PCR shows the expression levels of senescence markers were significantly reduced in rapamycin-treated rBMSCs (*n* = 3), *p < 0.05;**p < 0.01 by Student *t* test. **h** 1 × 10^6^ primary BMSCs derived from control or D-Gal-treated rats (p3-p5) were treated with rapamycin for 48 h. Representative Western blot results shows that rapamycin treatment suppresses p70S6K activation and upregulates the expression of H3K9me3 in rBMSCs derived from control and D-Gal-treated rats (*n* = 4)

To further validate the interplay between mTORC1 pathway and heterochromatin organization in vivo, we established the accelerated aging model in rats using D-galactose (D-Gal). Four months of D-Gal injection significantly reduced the weight gain of rats (Fig. 6a), whereas it had no effect on bone length (Fig. 6b). Further mechanical tests on the control or D-gal-treated bone indicated that D-Gal treatment decreased the maximum force, ultimate stress, and Young’s modulus (Fig. 6c), suggesting that long time exposure to D-Gal impaired the physiological function of bone. Moreover, D-Gal-treated rats exhibited characteristics of osteoporosis as indicated by bone CT and morphometric indexes (Fig. 6d, e). Specifically, D-Gal treatment resulted in a global change of parameters indicative of bone mass loss, such as decreased bone volume, reduced trabecular number and thickness, and increased trabecular space (Fig. 6e). These results validate the inductive effect of D-Gal on bone aging. To further correlate these changes with bone marrow-derived MSCs (rBMSCs), we isolated rBMSCs from the femur of control or D-Gal-treated rats. As shown in Fig. 6f, D-Gal treatment dramatically activated mTORC1 pathway in rBMSCs, as the expression levels of mTORC1 and its downstream targets p70S6K and 4EBP were consistently upregulated. Meanwhile, the expression levels of H3K9me3 and HP1 γ and senescence marker p53 were significantly increased in D-Gal-treated rBMSCs. Additionally, the PDT of D-Gal-treated rBMSCs appears longer than that of control rBMSCs with no significant difference. These results indicate that rBMSCs derived from D-Gal-treated rats undergo senescence accompanying by activation of mTORC1 pathway and heterochromatin organization, which is consistent with the in vitro study. To further affirm the link between mTORC1 pathway and heterochromatin organization, we treated rBMSCs derived from either control- or D-Gal-administrated rats with rapamycin for 48 h. Intriguingly, rapamycin significantly reduced the expression of senescence markers (Fig. 6g) and increased the expression of H3K9me3 in rBMSCS, suggesting that rapamycin promotes heterochromatin organization and alleviates D-Gal-induced cellular senescence in rBMSCs.

## Discussion

The mTORC1 pathway is a fundamental nutritious pathway which senses both intracellular and extracellular signals and works as a central regulator of metabolism, growth, proliferation, and survival [7]. Recent studies have demonstrated that prolonged activation of mTORC1 pathway is closely associated with cellular senescence and premature aging [5, 15, 30, 31]. Inhibition of the mTORC1 with rapamycin is currently the only known pharmacological treatment that increases lifespan in all model organisms studied [7]. However, a number of fundamental questions remain unanswered regarding the mechanisms by which rapamycin modulates cellular senescence and age-related pathophysiology. In this study, we have identified a previously unrecognized role of mTORC1/p70S6K pathway in regulating heterochromatin organization, which plays a critical role in the control of SIPS.

By using two acute senescence models, we found that both Dox and H_2_O_2_ evoked a rapid activation of mTORC1 pathway (3–9 h after the end of treatment), long before hUC-MSCs exhibited senescence phenotypes (Fig. 1). On the other hand, Dox or H_2_O_2_ induced DNA damage response as early as 3 h after drug withdrawal and reached its peak at 24 h (Fig. 4a). Pretreatment with rapamycin dampened DNA damage starting from 3 h after the end of Dox or H_2_O_2_ treatment and alleviated SASP induction and senescence phenotype afterward (Fig. 2). In contrast, direct activation of mTORC1 by MHY 1485 aggravated Dox-induced DNA damage and senescence (Fig. 3c, d). Besides, we have identified p70S6K as the main downstream target of rapamycin during MSC senescence, as knockdown of p70S6K markedly reduced Dox-induced DNA damage and cellular senescence to the same extent as rapamycin did (Fig. 3a, b). Recently, several studies reported the importance of p70S6K in cellular senescence. For instance, senescence in fibroblasts induced by persistent mTORC1 activation depends on p70S6K, since p70S6K phosphorylates ZRF1 on Ser47 and increases the expression of p16 to induce cell cycle arrest, leading to fibroblast senescence [32]. Alternatively, Xie et al. reported that mTORC1/p70S6K pathway impaired DNA damage repair mechanism via phosphorylation of RNF168 which inhibited E3 ligase activity and increased DNA accumulation [33]. The communication between the DNA damage response and the mTORC1 pathway is one of the main strategies employed by the cell to face genotoxic and oxidative stress [34]. Given the rapid induction of DNA damage response by both Dox and H_2_O_2_ and the finding that mTORC1/p70S6K pathway regulates DNA damage response in advance of senescent characteristics, it is plausible that the regulatory role of mTORC1/p70S6K in SIPS of hUC-MSCs is attributed mainly to its effect on DNA damage response.

Cellular senescence is accompanied by extensive spatial rearrangement of heterochromatin; however, the physiological function of heterochromatin organization during this process is not clear. Some studies showed that in the early stage of cellular senescence, heterochromatin structure underwent dynamic remodeling via a striking transition from a permissive state to a restrictive state, which posed a barrier to DNA damage repair machinery leading to DNA damage accumulation [35, 36]. Alternatively, other studies demonstrated that pharmacological and genetic perturbation of heterochromatin increased DNA damage signaling, indicating heterochromatin restrains DNA damage response [37, 38]. In our study, we found that heterochromatin markers H3K9me3 and HP1γ were dramatically induced at 9 h after genotoxic or oxidative stress (Fig. 4a and Fig. S2B). In particular, while global expression levels of heterochromatin marks changed mildly, a spatial rearrangement of H3K9me3 constituted characteristic feature of SAHF in hUC-MSCs. The regulatory role of heterochromatin organization in SIPS was demonstrated by Chaetocin, which is a pan inhibitor for H3K9 methyltransferase. Interestingly, depletion of H3K9 methylation by Chaetocin led to intensified DNA damage response and increased cellular senescence in response to Dox (Fig. 4c, d), indicating heterochromatin perturbation leads to an increase of DNA damage response in genotoxic damage-induced senescence. Indeed, recent study showed that heterochromatin formation played a key role in lifespan, as decreased heterochromatin levels shortened lifespan, whereas increasing heterochromatin prolonged lifespan [39]. Additionally, several studies also revealed that the expression levels of heterochromatin marks were significantly reduced in bone marrow-derived MSCs of old individuals compared to young individuals [20, 23]. Thus, our results support a scenario in which stress-induced heterochromatin organization plays an important role in restraining DNA damage response, which can be achieved by confining the access of DNA damage sensors to DNA lesions, thus blocking the augmentation of DNA damage signaling and preventing SIPS progression [37].

An interesting finding from the current study is the interplay between the mTORC1/p70S6K pathway and the heterochromatin organization process during SIPS. We demonstrated that both rapamycin and p70S6K knockdown significantly promoted heterochromatin organization induced by Dox or H_2_O_2_ (Fig. 5 and Fig. S3), whereas activation of mTORC1 via MHY1485 mitigated the Dox-induced expression of H3K9me3 and HP1γ (Fig. 5d, e). In line with the cell models, the link between mTORC1 and heterochromatin changes was recapitulated in D-Gal-induced bone aging model, which demonstrated a concurrent activation of mTORC1 pathway and heterochromatin organization (Fig. 6f). Moreover, rapamycin treatment reversed D-Gal-induced senescence and upregulated heterochromatin marks in rBMSCs (Fig. 6g, h). While the molecular mechanism by which mTORC1/p70S6K regulates heterochromatin organization remains unclear, recent findings have demonstrated that nuclear mTOR protein can bind to thousands of sites in the genome, presuming that it may facilitate DNA damage signaling [40–42]. In addition, mTOR signaling has been reported to induce hypermethylation of different tumor suppressor genes via upregulating DNA methyltransferases and histone-modifying enzymes [43, 44]. In particular, previous study showed that suppression of PI3K/mTOR inhibited the expression of EHMT2, which is a H3K9 methyltransferase, therefore enhancing the therapeutic response in pancreatic cancer [45]. Based on these findings, it will be interesting to explore the possibility that mTORC1/p70S6K suppresses heterochromatin organization via H3K9-modifying enzymes. On the other hand, given the established link between mTORC1 pathway and human aging and our previous finding that heterochromatin organization functions as a protective mechanism against DNA damage along with MSC aging [20], it is explicable that along with human aging, prolonged activation of mTORC1 pathway suppresses heterochromatin organization, which adversely make these stem cells more susceptible to genotoxic damage.

## Conclusions

Collectively, we propose that inhibition of the mTORC1/p70S6K pathway promotes heterochromatin organization and maintains the steady state of heterochromatin, which may prevent DNA from further damage during stress-induced cellular senescence. This could be another underlying mechanism behind the anti-senescence effects of rapamycin or other methods to suppress mTORC1/p70S6K pathway, which adds new elements to our understanding of how the stress-induced DNA damage response and cellular senescence can be managed (Fig. S4).

## Supplementary information

**Additional file 1: Supplementary Fig. S1.** Knockdown of p70S6K ameliorates H_2_O_2_-induced senescence in hUC-MSCs. β-gal staining of control siRNA- or p70S6K-siRNA- treated hUC-MSCs in the presence or absence of H_2_O_2_. 1x10^5^ hUC-MSCs (p6-p7) were seeded in 6-well plates and transfected with siRNAs. 24 hours later, the cells were treated with H_2_O_2_ and collected 2 days afterward. Quantification data is shown below, Data are presented as the mean ± SEM. **p<0.01;***p<0.001 by One way Anova with Tukey’s post hoc test. **Supplementary Figure S2.** Heterochromatin organization is involved in Dox or H_2_O_2_-induced senescence. (**A**) Representative Western blot shows that the expression levels of heterochromatin marks were dramatically reduced at the late stage of senescence. 2x10^5^ hUC-MSCs (p5-p7) were seeded in 6-well plates and treated with Dox (10^-8^M) for 24h or H_2_O_2_(300nM) for 3h, then washed with PBS and grown for another 2-4 days. After that, the cell lysates were collected for Western blot; **(B)** Representative photos and quantification of immunofluorescence staining of H3K9me3 and HP1γ in H_2_O_2_-induced hUC-MSCs at different time points (scale bar=10μm). 1x10^4^ hUC-MSCs (p5-p7) were seeded on coverslip and treated with H_2_O_2_. The cells were washed with PBS and incubated in the fresh media for 24 hours. Quantification is shown at the right panel, mean±SEM of values from three independent experiments with triplicate wells analyzed on 6-8 cells/field from five different fields. *p<0.05; **p<0.01;***p<0.001 by One way Anova with Tukey’s post hoc test; **(C)** 2x10^5^ hUC-MSCs (p5-p7) were seeded in 6-well plates and treated with Dox with or without Chaetocin. The cells were collected 2 days afterward. β-Gal staining shows that Chaetocin aggravates Dox-induced hUC-MSC senescence (scale bar=100μm). **Supplementary Figure S3.** Rapamycin promotes heterochromatin organization in H_2_O_2_-induced senescence. 1x10^4^ hUC-MSCs (p8-p9) were seeded in coverslips and treated with H_2_O_2_ with or without Rapamycin. Cells were collected after 24 hours. Representative images and quantification of immunofluorescence staining of HP1γ and H3K9me3 in control or Rapamycin-treated hUC-MSCs in response to Dox (scale bar=10μm). Quantification is shown on the right panel, mean±SEM of values from three independent experiments with triplicate wells analyzed on 6-8 cells/field from five different fields.*p<0.05; **p<0.01 by One way Anova with Tukey’s post hoc test. **Supplementary Figure S4.** Illustration of the crosstalk between mTORC1/p70S6K pathway and heterochromatin organization in stress-induced senescence. Inhibition of the mTORC1/p70S6K pathway promotes heterochromatin reorganization and maintains the steady state of heterochromatin, which may prevent DNA from further damage during stress-induced cellular senescence. We propose this could be another underlying mechanism behind the anti-senescence effects of rapamycin or other methods to suppress mTORC1/p70S6K pathway. On the other hand, heterochromatin reorganization functions as an inherent and protective mechanism to restrain DNA damage and delay the senescence process. Impairment of this protective mechanism might further activates mTORC1 pathway to deepen the senescence process.

**Additional file 2: Supplementary Table 1**. Primers used in this study. **Supplementary Table 2**. The antibodies used in this study.

## Data Availability

The datasets used and/or analyzed during the current study are available from the corresponding author on reasonable request.

## Abbreviations

Dox: Doxorubicin
SASP: Senescence associated secretory phenotype
D-Gal: D-galactose
mTORC1: Mammalian target of rapamycin complex 1
p70S6K: Ribosomal protein S6 kinase
4E-BP1: Eukaryotic initiation factor 4E-binding protein 1
SIPS: Stress-induced premature senescence
SAHF: Senescence-associated heterochromatic foci
HGPS: Hutchinson-Gilford progeria syndrome HGPS
MSC: Mesenchymal stromal cells
hUCMSCs: Human umbilical cord-derived MSCs
hBM-MSCs: Human bone marrow-derived MSCs
