# Supplementary figures and images for: Functional crosstalk between mTORC1/p70S6K pathway and heterochromatin organization in stress-induced senescence of MSCs
Source: Stem Cell Res Ther. 2020 Jul 13;11:279. doi: 10.1186/s13287-020-01798-1 (PMC7359252; doi:10.1186/s13287-020-01798-1)

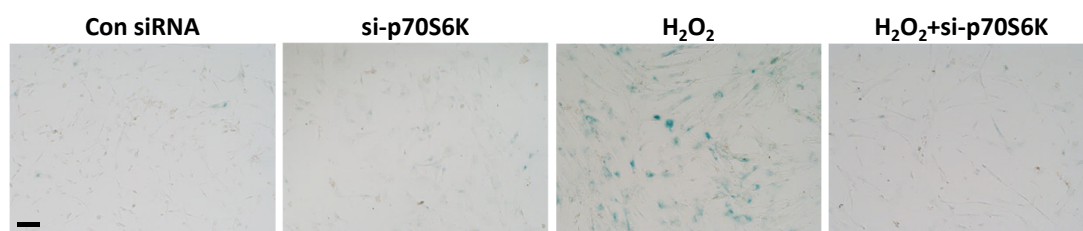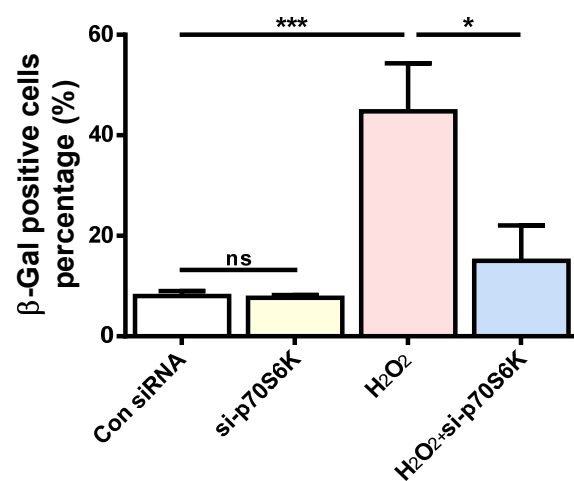

A

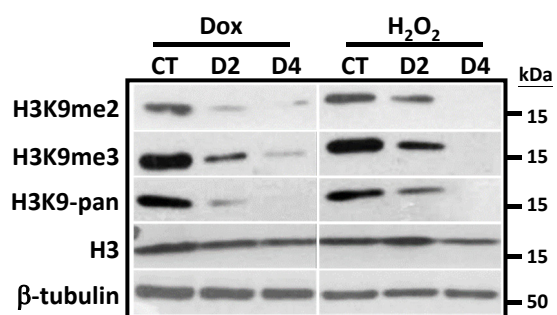

B

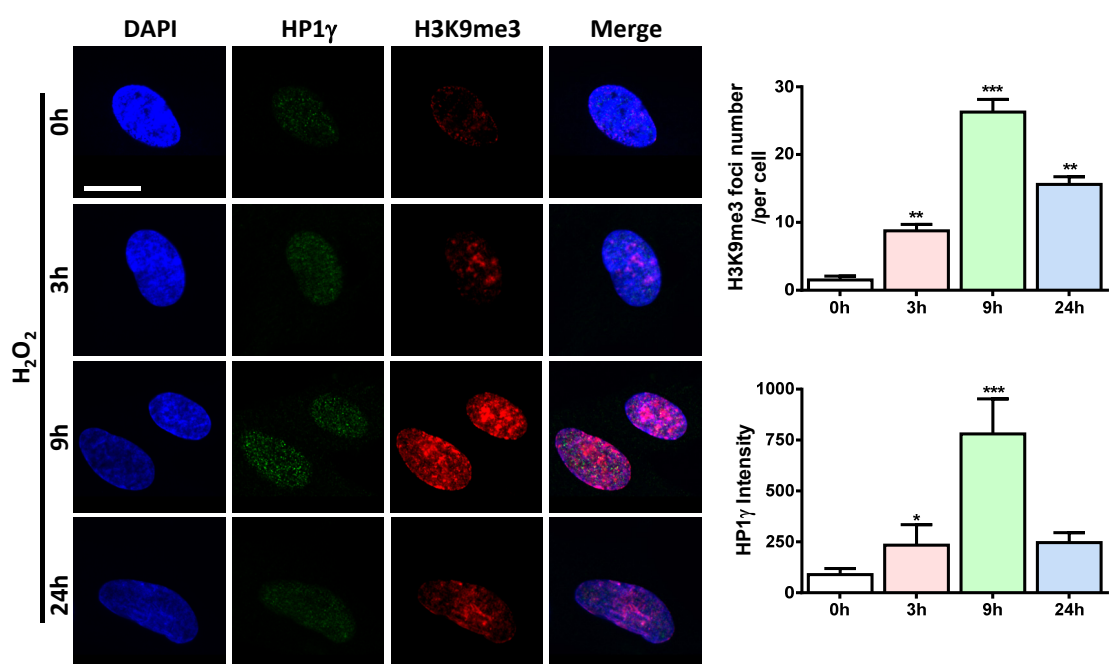

C

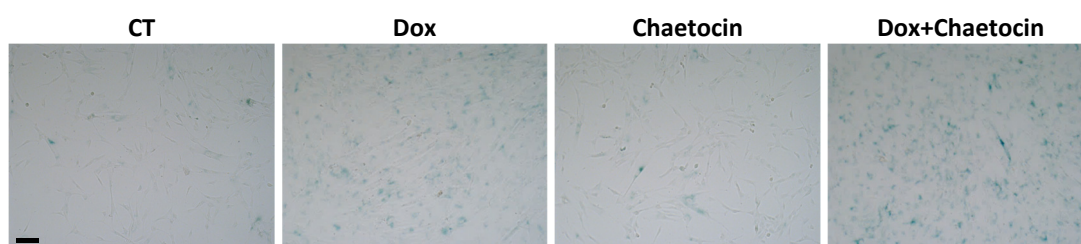

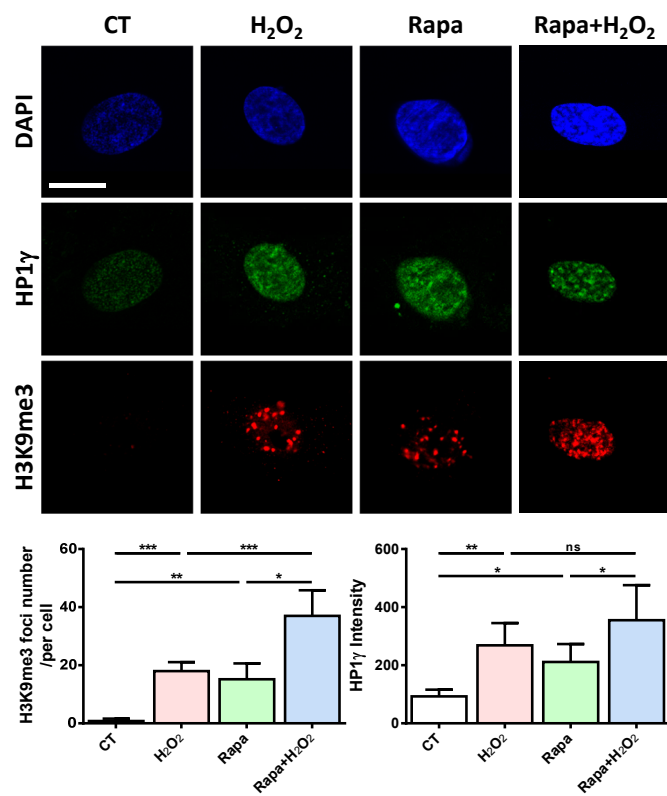

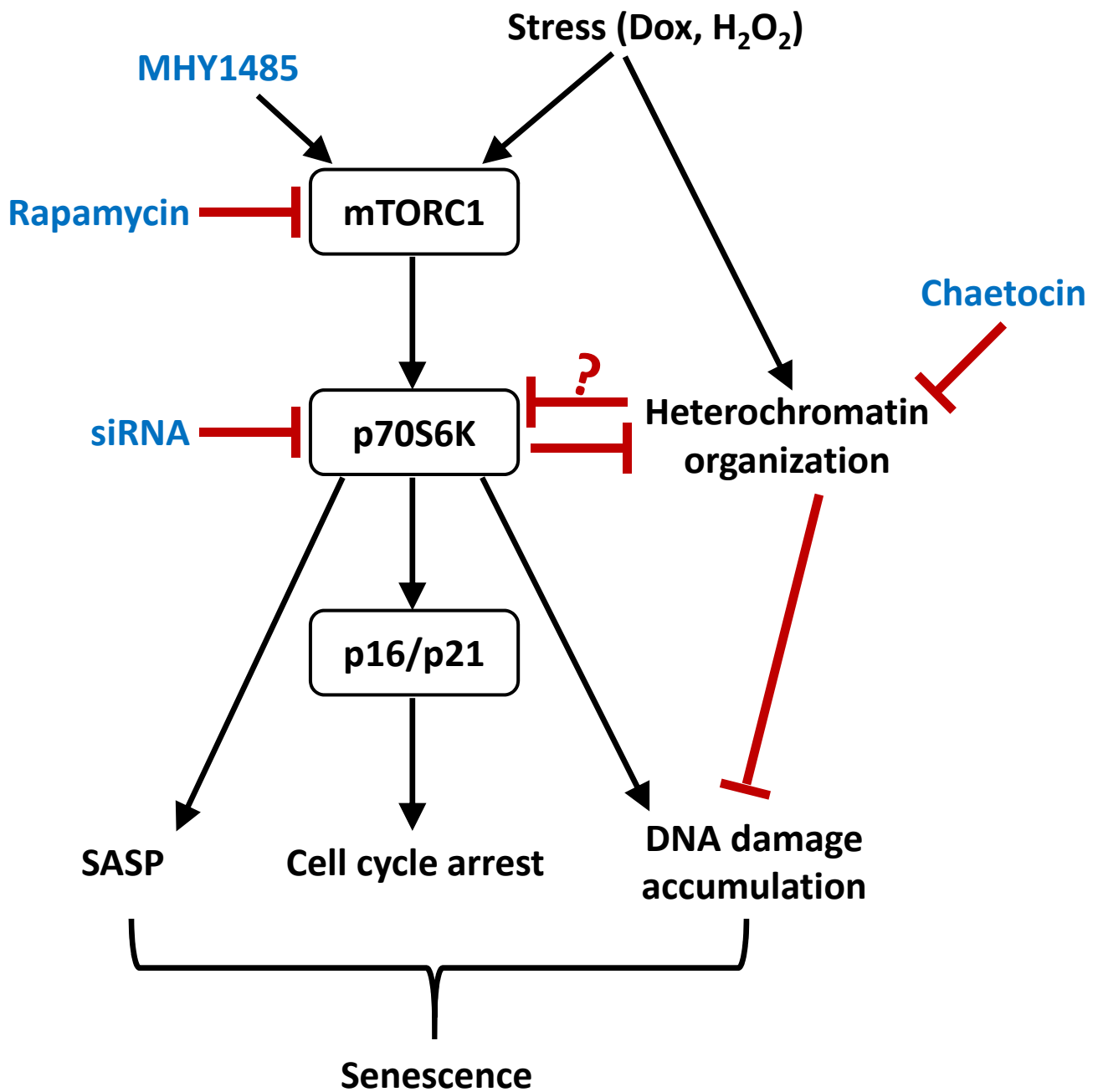

Supplement: Supplementary file 1 — Additional file 1: Supplementary Fig. S1. Knockdown of p70S6K ameliorates H2O2-induced senescence in hUC-MSCs. β-gal staining of control siRNA- or p70S6K-siRNA- treated hUC-MSCs in the presence or absence of H2O2. 1x10^5^ hUC-MSCs (p6-p7) were seeded in 6-well plates and transfected with siRNAs. 24 hours later, the cells were treated with H2O2 and collected 2 days afterward. Quantification data is shown below, Data are presented as the mean ± SEM. **p<0.01;***p<0.001 by One way Anova with Tukey’s post hoc test. Supplementary Figure S2. Heterochromatin organization is involved in Dox or H2O2-induced senescence. (A) Representative Western blot shows that the expression levels of heterochromatin marks were dramatically reduced at the late stage of senescence. 2x10^5^ hUC-MSCs (p5-p7) were seeded in 6-well plates and treated with Dox (10-8M) for 24h or H2O2(300nM) for 3h, then washed with PBS and grown for another 2-4 days. After that, the cell lysates were collected for Western blot; (B) Representative photos and quantification of immunofluorescence staining of H3K9me3 and HP1γ in H2O2-induced hUC-MSCs at different time points (scale bar=10μm). 1x10^4^ hUC-MSCs (p5-p7) were seeded on coverslip and treated with H2O2. The cells were washed with PBS and incubated in the fresh media for 24 hours. Quantification is shown at the right panel, mean±SEM of values from three independent experiments with triplicate wells analyzed on 6-8 cells/field from five different fields. *p<0.05; **p<0.01;***p<0.001 by One way Anova with Tukey’s post hoc test; (C) 2x10^5^ hUC-MSCs (p5-p7) were seeded in 6-well plates and treated with Dox with or without Chaetocin. The cells were collected 2 days afterward. β-Gal staining shows that Chaetocin aggravates Dox-induced hUC-MSC senescence (scale bar=100μm). Supplementary Figure S3. Rapamycin promotes heterochromatin organization in H2O2-induced senescence. 1x10^4^ hUC-MSCs (p8-p9) were seeded in coverslips and treated with H2O2 with or without Rapamycin. Ce [file 13287_2020_1798_MOESM1_ESM.pdf]
