# Supplementary material for: Functional crosstalk between mTORC1/p70S6K pathway and heterochromatin organization in stress-induced senescence of MSCs
Source: Stem Cell Res Ther. 2020 Jul 13;11:279. doi: 10.1186/s13287-020-01798-1 (PMC7359252; doi:10.1186/s13287-020-01798-1)
Supplement: Supplementary file 2 — Additional file 2: Supplementary Table 1. Primers used in this study. Supplementary Table 2. The antibodies used in this study. [file 13287_2020_1798_MOESM2_ESM.pdf]

Supplementary Table 1: Primers used in this study

Primers for qPCR

Target	Forward primer (5' to 3')	Reverse primer (5' to 3')
h p15	GGGGACTAGTGGAGAAGGTG	GGTGAGAGTGGCAGGGTC
h p16	CTTCCTGGACACGCTGGT	CATGGTTACTGCCTCTGGTG
h p21	CGACTGTGATGCGCTAATGG	CTGCCTCCTCCCAACTCATC
h IL-1α	AGATGCCTGAGATACCCAAAACC	CCAAGCACACCCAGTAGTCT
h IL-1β	ATGATGGCTTATTACAGTGGCAA	GTCGGAGATTCGTAGCTGGA
h IL-6	ACTCACCTCTTCAGAACGAATTG	CCATCTTTGGAAGGTTCAGGTTG
h Ccl-2	CAGCCAGATGCAATCAATGCC	CAGCCAGATGCAATCAATGCC
h Cxcl-10	GTGGCATTCAAGGAGTACCTC	GCCTTCGATTCTGGATTCAG
h p53	GAGGTTGGCTCTGACTGTACC	TCCGTCCCAGTAGATTACCAC
r p15/INK4b	TCACCAGACCTGTGCATGAT	AGGCGTCACACACATCCAG
r p16/INK4a	ACCAAACGCCCCGAACA	GAGAGCTGCCACTTTGACGT
r p21/CIP1	CAGCCACAGGCACCATGTC	ACAGACGACGGCATACTTTGC
r p53	CCCAGGGAGTGCAAAGAGAG	TCTCGGAACATCTCGAAGCG

Supplementary Table 2: The antibodies used in this study

**Antibody name**	**Company**	**Catalogue number**
mTOR	Cell Signaling Technology	Cat.No.2972S
mTOR	Millipore	Cat. No. 04-385
phospho-mTOR	Abcam	Cat.No.ab109268
phospho-mTOR	Cell Signaling Technology	Cat.No.5536S
p70s6k	Cell Signaling Technology	Cat.No.9202S
phospho-p70s6k	Cell Signaling Technology	Cat.No.9234S
4EBP1	Cell Signaling Technology	Cat.No.#9644
phosph-4EBP1	Cell Signaling Technology	Cat.No.2855
Tubulin	Santa Cruz	Cat.No.sc-5274
H3K9me3	Cell Signaling Technology	Cat.No.#13969
H3K9me2,3	Cell Signaling Technology	Cat.No.5327
HP1-γ	Merck Millipore	Cat.No.MABE656
Phosphor-H_2A.X	Cell Signaling Technology	Cat.No.#2577
